# Supplementary material for: Diurnal changes in the efficiency of information transmission at a sensory synapse
Source: Nat Commun. 2022 May 12;13:2613. doi: 10.1038/s41467-022-30202-0 (PMC9098879; doi:10.1038/s41467-022-30202-0)
Supplement: Supplementary file 1 — Supplementary Information [file 41467_2022_30202_MOESM1_ESM.pdf]

Diurnal changes in the efficiency of information transmission at a sensory synapse

José Moya-Díaz, Ben James, Federico Esposti, Jamie Johnston and Leon Lagnado*

Supplementary Information (Figures S1-S4)

Supplementary Figure 1

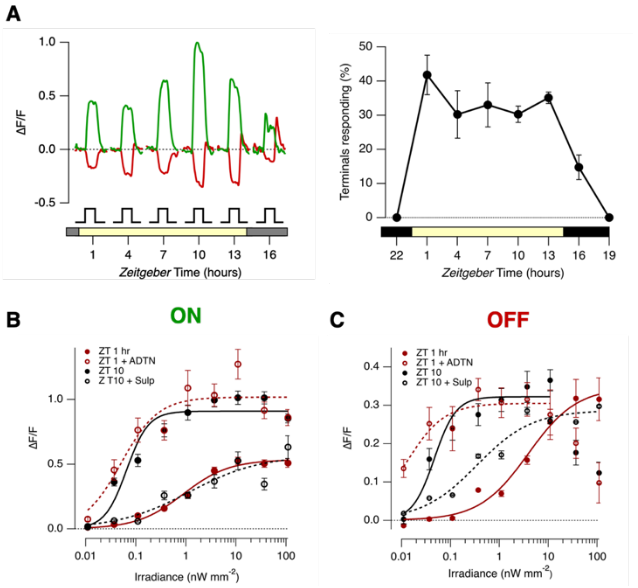

Figure S1. Diurnal changes in luminance sensitivity are co-ordinated by dopamine

A. Left: Averaged SyGCaMP2 signals at different *Zeitgeber* time. ON terminals green (n = 535 from 38 fish) and OFF terminals red (n = 355 from 38 fish). Each step of light (10 nW mm^{-2}) lasted 3 s. These averages are only from responsive terminals. Right: the percentage of terminals generating a significant response to the same light step (averaged across both ON and OFF). Bars show SD. **B.** Effects of manipulating dopamine signalling on luminance sensitivity of the ON channel. Luminance vs. response plots for ON terminals. Red circles compare this function at ZT 1 hr under control conditions (solid circle) and after injection of the dopamine receptor agonist ADTN (~0.2 µM; open circles). ADTN caused a prompt change in the luminance-response function to forms measured at ZT 10 hrs (solid black circles), increasing R_{max} from 0.53 ± 0.02 to 1.02 ± 0.07, and reducing $I_{1/2}$ from 0.88 ± 0.18 nW mm^{-2} to 0.05 ± 0.02 nW mm^{-2} (± sd, as estimated from the fitted Hill function shown). Each point shows the mean ± s.e.m. The higher gain and luminance sensitivity at ZT 10 hrs could be explained as an effect of dopamine at D2 receptors, because it was completely reversed by injection of the selective D2 receptor antagonist sulpiride (~2 µM; open black circles; R_{max} = 0.57 ± 0.13, $I_{1/2}$ = 1.16 ± 1.34 nW mm^{-2}). Results collected from *n* = 535 terminals from 38 fish. **C.** Effects of manipulating dopamine signalling on luminance sensitivity of the OFF channel. Comparing control responses at ZT 1 hr and 10 hrs showed a significant reduction in $I_{1/2}$ from 3.9 ± 1.3 to 0.0128 ± 0.005, but *without* a significant change in R_{max} (0.35 ± 0.03 vs 0.30 ± 0.02). ADTN injected at ZT 1 caused a prompt increase in luminance sensitivity, reducing $I_{1/2}$ to 0.013 ± 0.005 nW mm^{-2}. The higher luminance sensitivity at ZT 10 hrs could be partly explained as an effect of dopamine at D2 receptors, because injection of sulpiride (~2 µM; open black circles) increased $I_{1/2}$ from 0.05 ± 0.01 nW mm^{-2} to 0.35 ± 0.14 nW mm^{-2}. Results from *n* = 355 terminals from 38 fish. Each point shows the mean ± s.e.m. Source data are provided as a Source Data file.

Supplementary Figure 2

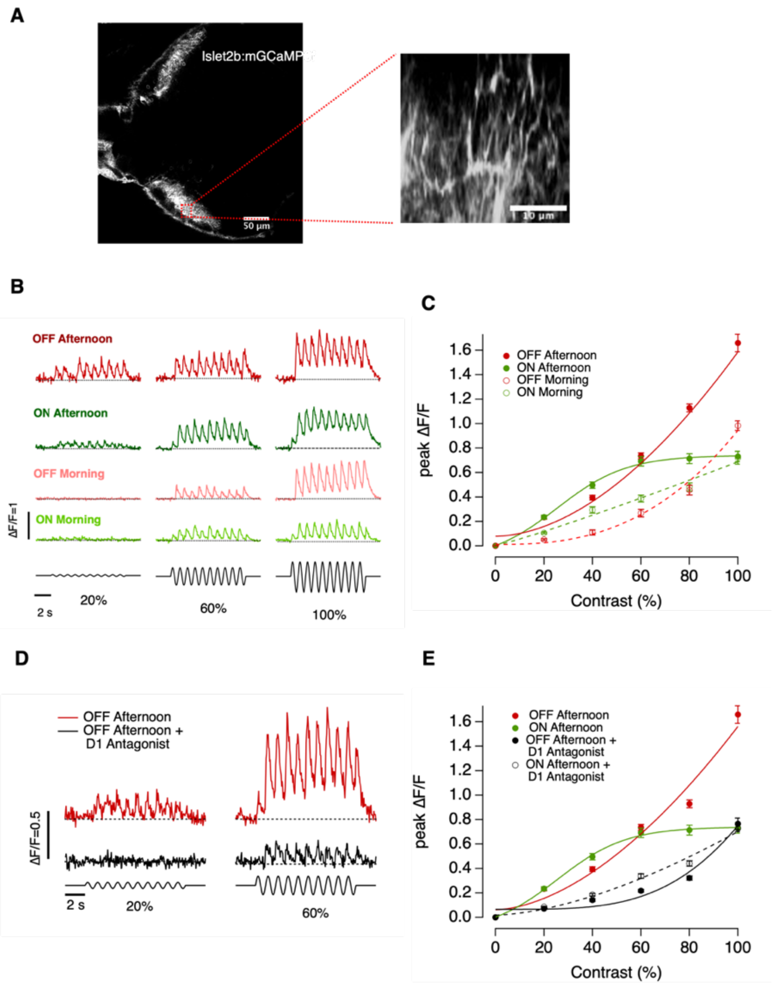

Figure S2. Diurnal changes in the visual signal delivered to the optic tectum

A. *Left panel*. Multiphoton section through the tectum of a zebrafish larva islet2b:mGCaMP6f (7 dpf) expressing the calcium reporter mGCaMP6f, which labels axons and synaptic terminals of retinal ganglion cells (RGCs). *Right panel*. Blow-up of the square red shown in the left panel. The image shows the Z plane reconstruction from where the synaptic responses were recorded. **B.** mGCaMP6f signals from individual ON and OFF RGCs synapses elicited using a stimulus contrast of 20%,60% and 100 %. Note that here the stimulus was modulated at 1 Hz (full field, sine wave) rather than the 5 Hz used in the main body of the paper. **C.** Average contrast-response functions displayed by ON and OFF RGCs, where the response (*R*) was quantified as the average of the fluorescence peak amplitudes measured at each cycle of stimulation. Note the differences in the magnitude of the responses between OFF RGCs in the morning relative to afternoon. Each point shows the mean ± s.e.m. Number of RGCs was: ON afternoon, 14; OFF afternoon, 13; ON morning, 11; OFF morning, 12. **D.** mGCaMP6f signals from an individual OFF RGCs before and after intravitreal injection of the D1 antagonist in the afternoon. **E.** Average contrast-response functions RGCs before and after administration of the D1 antagonist SCH 23390 in the afternoon. Number of RGCs: 12 ON and 11O FF. Note the dramatic decrease in the magnitude of the response in both ON and OFF channels. Number of RGCs was: ON afternoon, 14; OFF afternoon, 13; ON morning, 11; OFF morning, 12. Each point shows the mean ± s.e.m. Source data are provided as a Source Data file.

Supplementary Figure 3

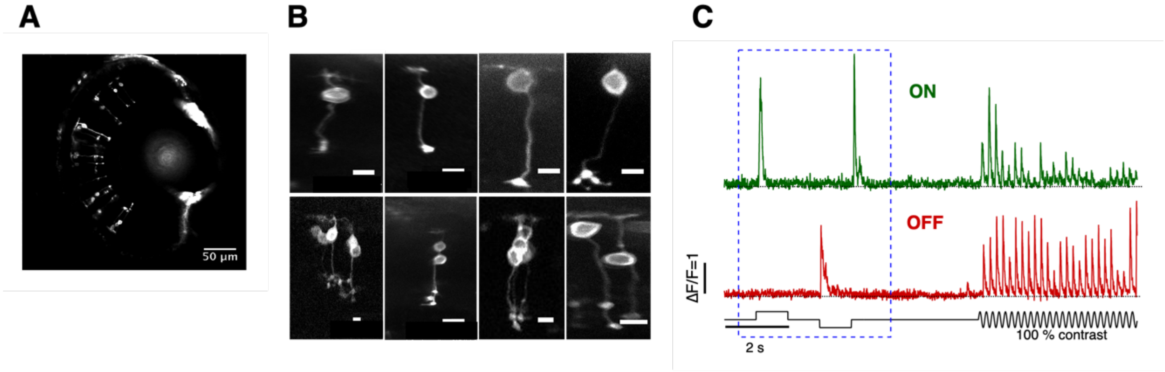

Figure S3. Examples of the different morphological types of bipolar cell sampled in this study.

A. Multiphoton section through the eye of a larval zebrafish (7 dpf) expressing iGluSnFr in a subset of bipolar cells. **B.** Examples of some of the different morphological subtypes of OFF (top) and ON (bottom) bipolar cell which were sampled in this study. All scale bars are 2 µm. **C.** We focused on a comparison on the two most basic functional types, ON and OFF cells, identified through their responses to steps of light applied before applying stimuli modulated at 5 Hz. iGluSnFR signals were measured in a total of 91 synapses in ON and 151 synapses in OFF cells.

Supplementary Figure 4

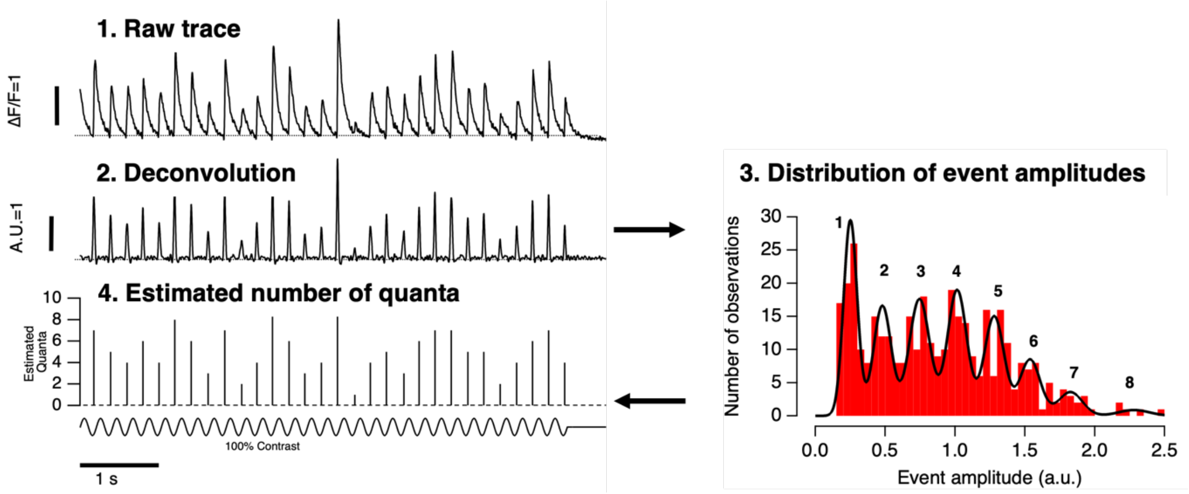

Figure S4. Decomposition of iGluSnFR signals into vesicle counts

Summary of the basic steps for quantal decomposition of iGluSnFr signals. Each event is assigned a time, t, and an estimated number of quanta, q, using the following basic steps.

1. Raw trace extracted from individual active zones (linescan, 1 KHz).

2. Trace deconvolved using the estimated Wiener filter and threshold crossings used to detect events above noise. The timing of the peak is the event time t. The amplitude of the event is the peak of the deconvolved trace at time t.

3. The distribution of event amplitudes is plotted and fitted by the sum of Gaussians with peaks differing by integer multiples of a quantal value q. This example is a histogram of event amplitudes for an active zone in which 373 events were accumulated using stimulus contrasts of 20%, 60% and 100% and a frequency of 5 Hz. The black line is a fit of eight Gaussians, identified using a Gaussian mixture model. Note that the variance of successive Gaussians did not increase in proportion to the peak number. The first peak had a value of 0.24, and the distance between peaks averaged 0.25, indicating the existence of a quantal event equivalent to ~0.25.

4. Maximum-likelihood estimation of the number of quanta, q, in each event based on its amplitude in 2 and the distribution in 3.

These steps are explained and validated in much greater detail in James et al. (2019).
